# Recent Topics on Deep-Sea Microbial Communities in Microbes and Environments

**DOI:** 10.1264/jsme2.ME3404rh

**Published:** 2019-12-27

**Authors:** Ken Takai

**Affiliations:** 1 Institute for Extra-cutting-edge Science and Technology Avant-garde Research (X-star), Japan Agency for Marine-Earth Science and Technology (JAMSTEC) 2–15 Natsushima-cho, Yokosuka 237–0061 Japan

Deep ocean exploration was motivated by a series of scientific challenges to disprove previously widespread misleading ideas, such as that absence of life in ocean water or the seafloor at greater depths than certain limitations (5). It was also promoted by gradual advances in technologies for water, sediment, and animal sample recoveries, such as trawls, grabs (dredges), box and gravity cores, traps, and CTD-niskin samplers. Using these methods, the first animal specimens were successfully collected from the deep seafloor and the unequivocal existence of animals in deep ocean water and the seafloor was proven in the last century (5).

Considerably later than the discovery and perception of deep-sea animal populations, psychrophilic and piezophilic microbial (bacterial) populations were identified in deep ocean sediments (27, 28). In more than 60 years of research, microbiological explorations have renewed our view of deep-sea microbial ecosystems. Deep-sea microbial ecosystems were initially considered to be static habitats and host to less abundant communities composed of minor extremophilic and long-surviving populations from the overlying ocean environments. However, they are now being recognized as dynamic habitats and host to more abundant communities composed of genetically diverse and functionally active populations that respond to variable oceanographic, geological, and geochemical events and impacts. In the past several years, many studies published in *Microbes and Environments* have attempted to elucidate the compositional and functional diversities of microbial communities in investigations of previously unexplored deep-sea biotopes and microbial cellular and molecular components.

Extremophilic microbial communities in deep-sea hydrothermal and seepage environments have been the most extensively studied among various deep-sea biotopes since their first discovery in 1977 (1). The compositional and functional diversities of chemosynthetic microbial communities and the molecular and energetic bases of community development have recently been reviewed (*e.g.*, 10, 12). However, it is important to note that the majority of previous microbiological investigations on chemosynthetic microbial communities associated with hydrothermal vents and chimney structures were only based on cultivation and gene amplicon sequencing techniques. Muto and colleagues successfully developed a simple and efficient RNA extraction method from deep-sea hydrothermal vent chimney structures (11). Using this new technique, further transcriptomic approaches will be applicable to diverse microbial communities associated with various hydrothermal mineral deposits, and the energetic and metabolic dynamics of chemosynthetic microbial community development will be addressed in the future using an RNA-based approach. Kato and colleagues were the first to report the biogeographical and intra-structural distribution of microbial communities in deep-sea ferromanganese (Fe-Mn) crusts at different depths of the Takuyo-Daigo Seamount (7). Although the growth mechanisms and biological contribution of Fe-Mn crusts remain unclear, this study indicated that the surface zones of Fe-Mn crusts are suitable habitats for specific microbial populations at different depths and may be host to as-yet-uncertain, but potentially significant mineral-microbial biogeochemical processes (7). Kato and colleagues also conducted a metagenomic analysis of microbial communities dwelling in metal sulfide deposits not directly associated with ongoing hydrothermal fluid activities (8). They found four new metagenome-assembled genomes (MAGs) within “*Candidatus* Hydrothermaarchaeota” (6), originally proposed as Marine Benthic Group E (MBGE) (19), and reported the metabolic versatility of previously uncultivated archaeal populations in hydrothermal environments.

Sediments and water columns are two major habitats of deep-sea microbial communities. A number of bacterial strains have been isolated and described from deep-sea sediments (2). However, the majority of oceanfloor sediments (abyssal plain sediments) are static and energetically and nutritionally barren, and genetically diverse and functionally active sedimentary microbial communities are present in the temporally and spatially limited hot spots of deep-sea sediments. However, the unexpectedly high activities of microbial carbon mineralization in hadal (>6,000 m) trench sediments renewed the concept of a ‘quiet deep-sea microbial ecosystem’ (3, 9, 23). Comparisons with the nearest abyssal plain sediments revealed that hadal trench sediments had greater or similar organic carbon contents and microbial cellular abundance, as well as higher O_2_ respiration activities in many of the hadal trenches (3, 9, 23). Nunoura and colleagues demonstrated that compositionally and functionally unique microbial communities specifically occur in hadal trench water columns and sediments, which may be distinguished from microbial communities in the overlying and surface waters and sediments, and this was supported by their recent findings on sedimentary microbial communities in the deepest Mariana Trench bottom published in *Microbes and Environments* (13–16). In addition, Hirai and colleagues reported improvements in the basic library construction technique using subnanograms of DNA assemblages obtained from low-biomass and volume-limited deep-sea water and sediment samples for ongoing and future metagenome-based microbiological investigations (4). On the other hand, based on elaborate radioisotope tracer experiments, Yanagawa and colleagues were the first to show methanol-driven microbial functions in subseafloor sediments (24). These findings revealed a significant role for methanol in biogeochemical processes, such as methane production, in spatially expansive, anaerobic deep-sea and subseafloor sediments.

Planktonic and benthic free-living microbial populations as well as symbiotic populations are of great interest in studies on deep-sea microbial ecosystems. Watsuji and colleagues investigated the epibiotic microbial communities of the deep-sea vent-endemic squat lobster *Shinkaia crosnieri*, which dominates in the chemosynthetic animal communities of the Okinawa Trough deep-sea hydrothermal systems (20–22). In a recent study published in *Microbes and Environments*, they showed that the epibiotic microbial community composition and metabolic functions varied in response to a shift in energy source availability during the laboratory rearing of host animals (20). These findings support the energetic control of chemosynthetic microbial community development in deep-sea hydrothermal environments proposed by Nakamura and Takai (12).

Deep-sea virospheres have been attracting increasing interest. Viral abundance and diversity in various deep-sea waters and sediments have been intensively investigated using metagenomic analyses of viromes (25, 26). However, viruses that infect deep-sea animals have not yet been identified. Using the unique extraction method of viral RNA assemblages called FLDS (17), Urayama and colleagues reported the RNA viral genome sequence latent in the cells of the deep-sea bone-worm *Osedax japonicus*, which thrives on fallen whole bones (18). This virus may represent a novel viral family from already known viral sequences (18). By applying the new method to different types of habitats and samples, including deep-sea animals and other eukaryotes, previously unidentified viral components and virospheres in the deep-sea environments will be clarified.

Deep-sea microbiology has not been extensively reported in *Microbes and Environments*. As the Editor-in-Chief and a deep-sea microbiologist, I hope that *Microbes and Environments* will become an attractive medium for a wide spectrum of research on deep-sea microbiology.

## References

[1] Corliss J.B., Dymond J., Gordon L.I. (1979). Submarine thermal springs on the Galapagos Rift. Science.

[2] Fang J., Zhang L., Bazylinski D.A. (2010). Deep-sea piezosphere and piezophiles: geomicrobiology and biogeochemistry. Trends Microbiol.

[3] Glud R.N., Wenzhöfer F., Middelboe M., Oguri K., Turnewitsch R., Canfield D.E., Kitazato H. (2013). High rates of microbial carbon turnover in sediments in the deepest oceanic trench on Earth. Nat Geosci.

[4] Hirai M., Nishi S., Tsuda M., Sunamura M., Takaki Y., Nunoura T. (2017). Library construction from subnanogram DNA for pelagic sea water and deep-sea sediments. Microbes Environ.

[5] Jamieson A. (2015). The Hadal Zone: Life in the Deepest Oceans.

[6] Jungblush S.P., Amend J.P., Rappe M.S. (2017). Metagenome sequencing and 98 microbial genomes from Juan de Fuca Ridge flank subsurface fluids. Sci Data.

[7] Kato S., Okumura T., Uematsu K., Hirai M., Iijima K., Usui A., Suziki K. (2018). Heterogeneity of microbial communities on deep-sea ferromanganese crusts in the Takuyo-Daigo Seamount. Microbes Environ.

[8] Kato S., Nakano S., Kouduka M., Hirai M., Suzuki K., Itoh T., Ohkuma M., Suzuki Y. (2019). Metabolic potential of as-yet-uncultured archaeal lineages of *Candidatus* Hydrothermarchaeota thriving in deep-sea metal sulfide deposits. Microbes Environ.

[9] Luo M., Glud R.N., Pan B., Wenzhöfer F., Xu Y., Lin G., Chen D. (2018). Benthic carbon mineralization in hadal trenches: insights from in situ determination of benthic oxygen consumption. Geophys Res Lett.

[10] McGlynn S.E. (2017). Energy metabolism during anaerobic methane oxidation in ANME Archaea. Microbes Environ.

[11] Muto H., Takaki Y., Hirai M., Mino S., Sawayama S., Takai K., Nakagawa S. (2017). A simple and efficient RNA extraction method from deep-sea hydrothermal vent chimney structure. Microbes Environ.

[12] Nakamura K., Takai K. (2014). Theoretical constraints of physical and chemical properties of hydrothermal fluids on variations in chemolithotrophic microbial communities in seafloor hydrothermal systems. Prog Earth Planet Sci.

[13] Nunoura T., Nishizawa M., Kikuchi T. (2013). Molecular biological and isotopic biogeochemical prognoses of the nitrification-driven dynamic microbial nitrogen cycle in hadopelagic sediments. Environ Microbiol.

[14] Nunoura T., Takaki Y., Hirai M. (2015). Hadal biosphere: insight into the microbial ecosystem in the deepest ocean on Earth. Proc Natl Acad Sci USA.

[15] Nunoura T., Hirai M., Yoshida-Takashima Y. (2016). Distribution and niche separation of planktonic microbial communities in the water columns from the surface to the hadal waters of the Japan trench under the eutrophic ocean. Front Microbiol.

[16] Nunoura T., Nishizawa M., Hirai M. (2018). Microbial diversity in sediments from the bottom of the Challenger Deep, the Mariana Trench. Microbes Environ.

[17] Urayama S.-I., Takaki Y., Nunoura T. (2016). FLDS: A comprehensive dsRNA sequencing method for intracellular RNA virus surveillance. Microbes Environ.

[18] Urayama S.-I., Takaki Y., Nunoura T., Miyamoto N. (2018). Complete genome sequence of a novel RNA virus identified from a deep-sea animal, *Osedax japonicus*. Microbes Environ.

[19] Vetriani C., Jannasch H.W., MacGregor B.J., Stahl D.A., Reysenbach A.L. (2001). Population structure and phylogenetic characterization of marine benthic archaea in deep-sea sediments. Appl Environ Microbiol.

[20] Watsuji T.-o., Nakagawa S., Tsuchida S., Toki T., Hirota A., Tsunogai U., Takai K. (2010). Diversity and function of epibiotic microbial communities on the galatheid crab, *Shinkaia crosnieri*. Microbes Environ.

[21] Watsuji T.-o.,  Yamamoto A., Motoki K., Ueda K., Hada E., Takaki Y., Kawagucci S., Takai K. (2015). Molecular evidence of digestion and absorption of epibiotic bacterial community by deep-sea crab *Shinkaia crosnieri*. ISME J.

[22] Watsuji T.-o., Motoki K., Hada E. (2018). Compositional and functional shifts in the epibiotic bacterial community of *Shinkaia crosnieri* Baba & Williams (a squat lobster from hydrothermal vents) during methane-fed rearing. Microbes Environ.

[23] Wenzhofer F., Oguri K., Middelboe M., Turnewitsch R., Toyofuku T., Kitazato H., Glud R.N. (2016). Benthic carbon mineralization in hadal trenches: Assessment by in situ O_2_ microprofile measurements. Deep Sea Res, Part I.

[24] Yanagawa K., Tani A., Yamamoto N., Hachikubo A., Kano A., Matsumoto R., Suzuki Y. (2016). Biogeochemical cycle of methanol in anoxic deep-sea sediments. Microbes Environ.

[25] Yoshida M., Takaki Y., Eitoku M., Nunoura T., Takai K. (2013). Metagenomic analysis of viral communities in (hado) pelagic sediments. PLoS One.

[26] Yoshida M., Mochizuki T., Urayama S.-I. (2018). Quantitative viral community DNA analysis reveals the dominance of single-stranded DNA viruses in offshore upper bathyal sediment from Tohoku, Japan. Front Microbiol.

[27] Zobell C.E., Morita R.Y. (1957). Barophilic bacteria in some deep sea sediments. J Bacteriol.

[28] Zobell C.E., Morita R.Y. (1959). Deep-sea bacteria. Galathea Report, Copenhagen.

